# Berberine Attenuates Development of the Hepatic Gluconeogenesis and Lipid Metabolism Disorder in Type 2 Diabetic Mice and in Palmitate-Incubated HepG2 Cells through Suppression of the HNF-4α miR122 Pathway

**DOI:** 10.1371/journal.pone.0152097

**Published:** 2016-03-24

**Authors:** Shengnan Wei, Ming Zhang, Yang Yu, Xiaoxin Lan, Fan Yao, Xin Yan, Li Chen, Grant M. Hatch

**Affiliations:** 1 Department of Pharmacology, College of Basic Medical Sciences, School of Nursing, Jilin University, Changchun, Jilin, China; 2 Department of Pharmacology & Therapeutics, Biochemistry and Medical Genetics, University of Manitoba, DREAM, Children’s Hospital Research Institute of Manitoba, Winnipeg, Manitoba, Canada; Boston University School of Medicine, UNITED STATES

## Abstract

Berberine (BBR) has been shown to exhibit protective effects against diabetes and dyslipidemia. Previous studies have indicated that BBR modulates lipid metabolism and inhibits hepatic gluconeogensis by decreasing expression of Hepatocyte Nuclear Factor-4α (HNF-4α). However, the mechanism involved in this process was unknown. In the current study, we examined the mechanism of how BBR attenuates hepatic gluconeogenesis and the lipid metabolism alterations observed in type 2 diabetic (T2D) mice and in palmitate (PA)-incubated HepG2 cells. Treatment with BBR for 4 weeks improve all biochemical parameters compared to T2D mice. Treatment of T2D mice for 4 weeks or treatment of PA-incubated HepG2 cells for 24 h with BBR decreased expression of HNF-4α and the microRNA miR122, the key gluconeogenesis enzymes Phosphoenolpyruvate carboxykinase (PEPCK) and Glucose-6-phosphatase (G6Pase) and the key lipid metabolism proteins Sterol response element binding protein-1 (SREBP-1), Fatty acid synthase-1 (FAS-1) and Acetyl-Coenzyme A carboxylase (ACCα) and increased Carnitine palmitoyltransferase-1(CPT-1) compared to T2D mice or PA-incubated HepG2 cells. Expression of HNF-4α in HepG2 cells increased expression of gluconeogenic and lipid metabolism enzymes and BBR treatment or knock down of miR122 attenuated the effect of HNF-4α expression. In contrast, BBR treatment did not alter expression of gluconeogenic and lipid metabolism enzymes in HepG2 cells with knockdown of HNF-4α. In addition, miR122 mimic increased expression of gluconeogenic and lipid metabolism enzymes in HepG2 cells with knockdown of HNF-4α. These data indicate that miR122 is a critical regulator in the downstream pathway of HNF-4α in the regulation of hepatic gluconeogenesis and lipid metabolism in HepG2 cells. The effect of BBR on hepatic gluconeogenesis and lipid metabolism is mediated through HNF-4α and is regulated downstream of miR122. Our data provide new evidence to support HNF-4α and miR122 regulated hepatic gluconeogenesis and lipid metabolism as promising therapeutic targets for the treatment of T2D.

## Introduction

Type 2 diabetes mellitus (T2D) is a metabolic syndrome characterized by a high circulating blood glucose level and a disorder of lipid metabolism [1–3]. As a major insulin sensitive organ, the liver plays a key role in both glucose and lipid homeostasis. Extensive effort has been focused on the treatment of the hepatic gluconeogenic and lipid metabolism disorders associated with T2D [1,4,5]. Although several drugs are available for the treatment of T2D, their effect on correcting the dyslipidemia associated with the disease is not obvious. Therefore, drugs which could target both the hepatic gluconeogenesis as well as the lipid metabolism disorder of T2D would serve as promising therapies [6,7].

The crosstalk that exists between hepatic gluconeogenesis and lipid metabolic pathways remains largely unexplored. Hepatocyte Nuclear Factor-4α (HNF-4α) is a key transcriptional factor essential for differentiation of liver [8,9]. HNF-4α dysfunction has been observed in mature onset diabetes of the young, T2D, dyslipidemia, and the metabolic syndrome [10,11]. In liver-specific HNF-4α knockout mice HNF-4α was shown to play a critical role in gluconeogenesis [12]. It was previously demonstrated that HNF-4α promoted expression of the key gluconeogenesis enzymes including phosphoenolpyruvate carboxykinase (PEPCK) and glucose-6-phosphatase (G6Pase) via binding to cis-elements in their promoters [13]. In addition, HNF-4α was reported to be critical in the maintenance of hepatocyte differentiation and is a major *in vivo* regulator of genes involved in the control of lipid homeostasis [14].

Micro RNAs (miRNAs) are small non-coding RNA molecules which function in RNA silencing and post-transcriptional regulation of gene expression [15]. MiR-122 is a predominant microRNA in the liver and was shown to be regulated by HNF-4α in both Huh7 cells and in mouse liver [15]. MiR-122 has been implicated in several important aspects of liver pathobiology, including lipid metabolism, hepatocarcinogenesis, and HCV replication [16]. Inhibition of miR-122 in a mouse model of diet-induced obesity resulted in decreased plasma cholesterol levels and reduced expression of several hepatic lipogenic genes [17]. Thus, miR122 may be one of the downstream factors of the HNF-4α pathway that regulate glucose and lipid homeostasis in liver.

Berberine (BBR) is an isoquinoline alkaloid originally isolated from extracts of the Chinese herb Coptis chinensis (Huang lian) [18]. Interests in the therapeutic effects of BBR against both diabetes and dyslipidemia have emerged over the past two decades [19–22]. Treatment of hypercholesterolemic patients for 3 months with BBR was shown to reduce circulating plasma cholesterol levels through a statin-independent mechanism [23]. It was subsequently hypothesized that BBR could be applied in clinical settings as an alternative treatment for statin resistant dyslipidemia patients [24–26]. In palmitate-incubated NIT-1 pancreatic β cells BBR treatment inhibited intracellular accumulation of triacylglycerol through decreased lipogenesis and increased fat acid oxidation [27]. BBR treatment significantly reduced the levels of fasting blood glucose, cholesterol, and triglyceride and improved the abnormal glucose tolerance observed in type 2 diabetic rats. In addition, BBR inhibited the expression of nuclear factor HNF-4α, PEPCK and G6Pase in type 2 diabetic rats [13]. The above studies suggest that BBR might serve as an ideal candidate for the treatment of type 2 diabetes since it modulates both glucose and lipid homeostasis. However, the mechanism for this effect of BBR and whether a crosslink exists between the regulation of glucose and lipid metabolism was unknown. In the present study, we examined expression of miR122, HNF-4α, and key gluconoegenesis and lipid metabolism enzymes in the liver of type 2 diabetic mice and in palmitate-incubated HepG2 cells after BBR treatment. We show that BBR acts on the HNF-4α regulated miR122 pathway to inhibit gluconeogenic targets and key lipid biosynthesis regulatory factors.

## Materials and Methods

### Materials

BBR (purity quotient > 99.8%) and STZ were purchased from Sigma Chemicals Co., Ltd (St. Louis, MO, USA). Glucose, total cholesterol (TC), triglyceride (TG) diagnostic test kits were purchased from BioSino Bio-technology and Science Inc. (Beijing, China). High-density lipoprotein cholesterol (HDL-C) and low-density lipoprotein cholesterol (LDL-C) ELISA kits and aspartate aminotransferase (AST) and alanine aminotransferase (ALT) analysis kits were purchased from Tianjin Nine Tripods Medical & Bioengineering Co., Ltd. (Tianjin, China). Insulin ELISA Kit was purchased from Merck Millipore Corporation (Darmstadt, Germany). HNF-4α plasmid was purchased from GenePharma Co., Ltd (Suzhou, China). HNF4α siRNA was purchased from Santa Cruz Co., Ltd (Shanghai, China). miR-122 plasmid, miR122 mimic, miR122, miR156a and U6 primers and miR-122 inhibitor were purchased from Ribobio (Guangzhou, China). Lipofectamine 2000 was purchased from Invitrogen (Carlsbad, CA). miRNeasy Mini Kit was purchased from Qiagen (Hilden, Germany). The TransScript First-Strand cDNA Synthesis Super Mix was purchased from Transgen Biotech, (Beijing, China). The Fast Start Universal SYBR Green Master (ROX) was purchased from Roche (Hacienda, CA, USA). The mouse or rabbit polyclonal antibodies and secondary antibodies were purchased from Abcam (Cambridge, MA, USA). All other reagents were purchased from Beijing Chemical Factory (Beijing, China).

### Animal model

Male C57BL/6J mice (18–22g) were purchased from the Vital River Laboratory Animals Technology Co., Ltd (Beijing, China). Animals were housed in individual cages (humidity: 60 ± 5%, 12 h dark–light cycle with access to free drinking water). The mice were randomly divided into 2 groups. Control animals were fed standard diet (5% fat, 53% carbohydrate, 23% protein, with a total caloric value of 25 KJ). A second group of age-matched animals were fed high fat diet (22% fat, 48% carbohydrate, and 20% protein with a total caloric value of 43 KJ). The diets were provided by the Experimental Animals Center of Jilin University (Jilin, China). The Ethics Committee for the Use of Experimental Animals of Jilin University approved all procedures. Animals were feed with regular chow or high-fat diet for 4 weeks, animals feeding with high fat diet were intraperitoneally injected with Streptozotocin (STZ) dissolved in citrate buffer (pH 4.5) at a dose of 100 mg/kg body weight. Control animals feeding with regular chow were injected with the citrate buffer alone. After 4 weeks of injection, these animals were tested for fasting blood glucose (FBG) levels. STZ treated animals with FBG higher than 7.8 mmol/L were considered diabetic. Berberine treatment was administrated after mice were diagnosed as diabetic for another 4 weeks. Diabetic mice with or without berberine treatment were all feed high fat diet until sacrifice. The animals were then divided into 4 groups with 12 animals in each group: control (Control) untreated fed standard diet; diabetic mice without any drug treatment (DM); diabetic treated with low oral dose 40 mg/kg BBR (LB) or high oral dose 160 mg/kg BBR (HB). After 4 weeks of BBR treatment animals were fasted for 12 h and an oral glucose tolerance test (OGTT) was conducted. At the end of the study, fasting plasma was collected for measurement, of fasting insulin (FINS), fasting blood glucose (FBG), TG, TC, HDL-C, LDL-C, AST, and ALT. All operations were strictly in accordance with the relevant instructions from commercial kits. The liver was then excised and frozen immediately in liquid nitrogen and stored at −80°C until further analysis. The experimental procedures were carried out in accordance with the “Guide for the Care and Use of Laboratory Animals” (NIH Publication No. 85–23, National Academy Press, Washington, DC, revised 1996). The experiments were approved by the Animal Research Committee o College of Basic Medical Sciences, Jilin University (permit number, SYSK 2013–0005).

#### Determination of fasting blood glucose (FBG), fasting blood insulin (FINS), oral glucose tolerance test (OGTT) and biochemical parameters in blood samples

After a 12 h fast animals were orally gavaged with 2g/kg body weight of glucose dissolved in water. Blood samples were collected at 0, 30, 60 and 120 min for measurement of FBG levels by the glucose oxidase method [28]. After 4 weeks of treatment, mice were fasted for 12 h and blood collected from the orbital venous plexus and plasma separated by centrifugation at 3500g for 15 min. FINS was determined using the insulin ELISA kit according to the manufacturer’s instructions. The insulin sensitivity index (ISI) was calculated according to the fasting insulin and glucose concentration. The formulae for calculation of ISI was: Ln (fasting blood glucose×fasting insulin)^-1^. TC, TG, ALT, AST, HDL-C, LDL-C were measured in plasma samples by using the appropriate kit according to the manufacturer’s instructions.

#### Histological analysis

Liver tissue was fixed in 10% buffered formalin and embedded in paraffin sections. Four millimeter sections were cut and mounted on glass slides. After dehydration, the sections were stained with hematoxylin and eosin. An IX71 system biological microscope (Olympus) was used for morphological observation.

### Cell culture and Oligonucleotide transfections

Palmitatic acid in the vitro experiment is bonded with fatty acid free BSA. Lipid-containing media were prepared by conjugation of free BSA using a modified method described[29]. Briefly, the 0.1M sodium palmitate was mixed with 5% fatty acid-free BSA at 1:9 ratio and incubated for one hour in 37°C. Before experiment, the stock solution was diluted in the culture medium to the required concentration, adjusted to a pH value of 7.5, and filter sterilized.

HepG2 cells were cultured in Dulbecco’s modified Eagle’s medium (DMEM) containing 10% fetal bovine serum. Two days before experiments, HepG2 cells were plated in 6-well plates at 1 x 10^5^ cells per well. After grown to 60%–70% confluence, cells were incubated in the absence or presence of 0.3 mM PA or 0.3 mM PA with 10 μM BBR dissolved in DMEM (2ml/well) for 24 h. In some experiments, HepG2 cells were cultured with HNF-4α plasmid or HNF4α siRNA or HNF-4α plasmid combined with 50 nM of miR-122 inhibitor or miR-122 mimic transfected with Lipofectamine 2000 and incubated for 6 h, then treated plus or minus 10 μM BBR for a further 24 h. Cell medium was then collected and the cells washed with PBS. Total RNA was extracted and quantitative real-time polymerase chain reaction (RT-PCR) analysis was performed as described below.

#### miR-122 quantification

Isolation of serum RNA and quantification of the miR-122 levels were performed as described previously [30,31]. Total RNA was extracted from 200 μl of serum with the miRNeasy Mini Kit (Qiagen, Germany). The expression levels of miRNAs were directly normalized to miR156a or U6, which were exogenously added and considered as the reference miRNA [32]. Total RNA was extracted from 6-well culture plates by the TRIzol method. The cDNA synthesis was performed with 1 μg of total RNA using the TransScript First-Strand cDNA Synthesis Super Mix (Transgen Biotech, China) according to the manufacturer’s instructions. RT-PCR was performed on ABI 7300 using Fast Start Universal SYBR Green Master (ROX) as a double-stranded DNA-specific dye, according to the manufacturer’s instructions (Roche, USA). RT-PCR was performed in duplicate, and the mean results calculated. The results of quantitative RT-PCR values were given as negatives of the cycle threshold (CT) values which correlate with the amount of miRNA. The relative expression levels were calculated as 2^-[(Ct miR-122)–(Ct of miR-156a or U6)]^.

#### mRNA quantification

Total RNA was extracted from 100 mg frozen liver tissue by the TRIzol method. After total RNA was extracted, reverse-transcript PCR was performed using the reverse transcription kit. The expression level of mRNA was quantified with real-time PCR by using SYBR Green qPCR mix in a Real-Time PCR System (Roche, USA). PCR products were quantified fluorometrically using SYBR Green, normalized to the housekeeping gene GAPDH and fold-change expression was calculated by using Ct values in comparison with controls. The sense and antisense PCR primers used were generated from Sangon Biotech Co., Ltd. (Shanghai, China) and were as follows (Table 1):

**Table 1:** Primer sequence.

Target gene	sense	antisense
**HNF4α**	5’TTGAAAATGTGCAGGTGTTGAC3’	5’CAGAGATGGGAGAGGTGATCTG3’
**SREBP1**	5’CGATGCCCTGAGGCTCTTT3’	5’CAGGTCTTTGAGCTCCACAATCT3’
**Fas-1**	5’CCTGGATAGCATTCCGAACCT3’	5’AGCACATCTCGAAGGCTACACA3’
**ACCα**	5’CGCTCAGGTCACCAAAAAGAAT3’	5’GTCCCGGCCACATAACTGAT3’
**G6Pase**	5’ACATCCGGGGCATCTACAATG3’	5’AAAGAGATGCAGGCCCAA3’
**PEPCK**	5’AGCCTCGACAGCCTGCCCACGG3’	5’CCAGTTGACCAAAGGCTTTT3’
**CPT-1**	5’AGCCTCGACAGCCTGCCCACGG3’	5’CCAGTTGACCAAAGGCTTTT3’
**GAPDH**	5’CCATGGAGAAGGCTGGG3’	5’CAAAGTTGTCATGGATGACC3’

#### Western blot analysis

Total protein was extracted from liver tissue and HepG2 cells following the manufacturer's protocols (Beyotime Institute of Biotechnology, China). Protein concentration was measured using the bicinchoninic acid (BCA) assay (Thermo, BCA protein assay kit) (Burlington, ON, Canada) with bovine serum albumin as standard. 80–120 μg protein was separated in a 12% SDS polyacrylamide gel and electro transferred onto polyvinylidene difluoride (PVDF) membranes (Bio-Rad). Membranes were blocked with 5% (w/v) skim milk or 5% BSA for 2 h at room temperature and then incubated with mouse or rabbit polyclonal antibodies (HNF4α, 1:500; SREBP-1, 1:1000; CPT-1, 1:800; FAS-1, 1:1000; ACCα, 1:1000; p-ACCα, 1:1000; β-actin, 1:1000) with light shaking overnight at 4°C. The membranes were washed 3 times for 5 min each with 15 ml of TBST (10 mM Tris–HCl, 150 mM NaCl and 0.1% (v/v) Tween-20) and then incubated with secondary antibody (1:2000) at room temperature for 2 h. Protein was visualized with enhanced chemiluminescence and images were generated with a GENE Imaging system. The images were quantified using Image Analysis Software (Quantity One). β-actin was used as the loading control.

### Statistical analysis

All data were expressed as a mean ± standard error (SEM). All analyses were performed by One-way analysis of variance (ANOVA). Differences between groups were determined using Student’s t-test. A p value of less than 0.05 was considered statistically significant. All statistical analyses were carried out using Statistical Product and Service Solutions(SPSS)20.0.

## Results

### BBR treatment improves blood biochemical parameters and liver morphology in type 2 diabetic mice

**Fig 1:**
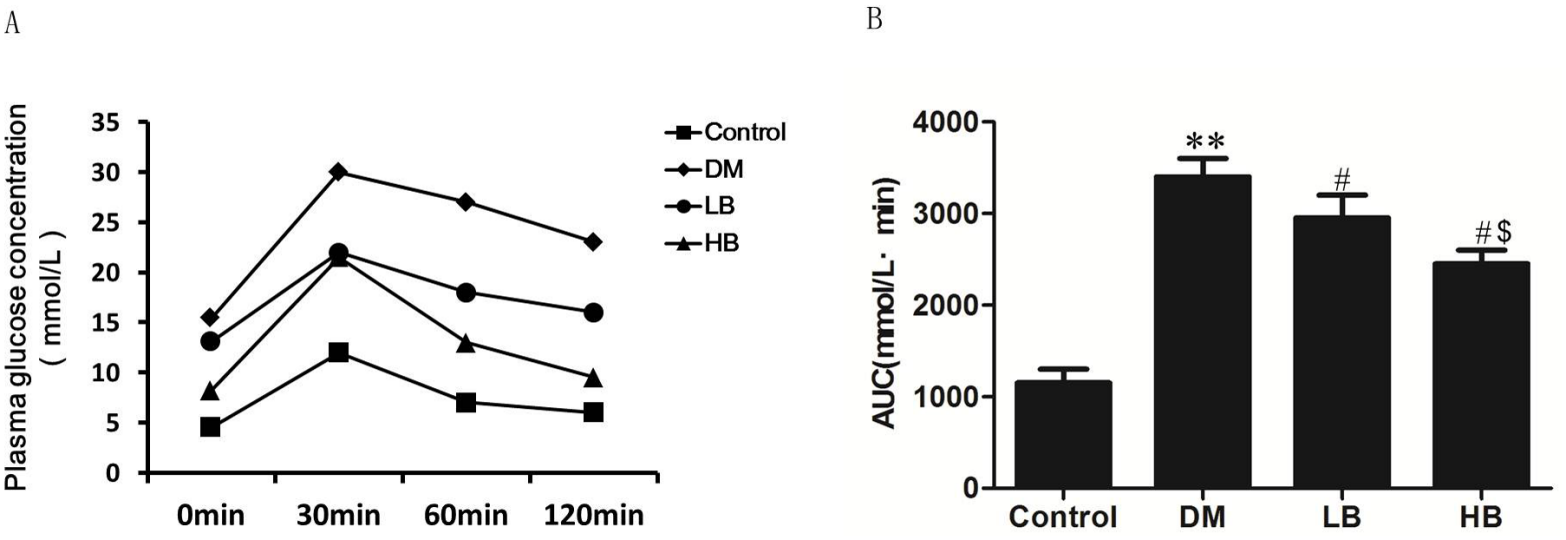
BBR treatment improves OGTT in diabetic mice. A. Plasma glucose concentrations were determined at 0, 30, 60 and 120 min after oral glucose administration of glucose as described in Materials and Methods. B. The area under the OGTT curve. Data are shown as the mean ± S.E.M (n = 12). **p<0.01 vs Control; ^#^p<0.05 vs DM; ^$^p<0.05 vs LB.

The blood biochemical parameters were examined in Control, DM, LB and HB animals. DM animals exhibited increased FBG, decreased FINS and an increased ISI indicative of insulin-resistance compared to controls (Table 2). The OGTT indicated that plasma glucose was elevated in DM animals and the area under the glucose concentration curve (AUC) was approximately 3-fold greater in DM animals compared to controls (Fig 1). Treatment of DM animals with BBR attenuated the elevated FBG, increased FINS levels and prevented the development of insulin resistance (Table 2). In addition, BBR treatment of DM animals attenuated the elevated plasma glucose and AUC (Fig 1). DM animals exhibited elevated plasma TC, TG and LDL-C and reduced HDL-C compared to controls (Table 2). Treatment of DM animals with BBR attenuated the elevated TC, TG and LDL-C and increased HDL-C. The higher dose of BBR (160mg/kg) in HB treated animals appeared more effective than the lower dose of BBR (40mg/kg) in LB treated animals.

**Table 2:** Biochemical parameters of diabetic mice treated with BBR.

Groups	Control	DM	LB	HB
N	12	12	12	12
FBG(mmol/L)	4.56±0.51	15.45±2.01*	12.12±0.75^#^	8.21±0.55^##^^Δ^
FINS(mU/L)	19.83±2.15	11.72±2.19*	14.12±1.03^#^	16.81±0.98^#^^Δ^
ISI	-4.20±0.52	-6.02±0.71*	-5.38±0.39^#^	-4.58±0.45^#^^Δ^
TC(mmol/L)	3.21±0.26	5.07±0.35**	4.66±0.31^#^	4.18±0.06^#^^Δ^
TG(mmol/L)	0.95±0.10	1.77±0.25**	1.13±0.10^#^	1.05±0.06^#^^Δ^
HDL-C(mmol/L)	4.32±0.52	2.76±0.31**	3.20±0.15^#^	3.71±0.17^##^^Δ^
LDL-C(mmol/L)	1.09±0.22	2.12±0.20*	1.81±0.32	1.62±0.43
ALT(U/L)	70.6±12.13	157.09±21.31*	123.32±10.68^#^	89.02±12.73^##^^Δ^
AST(U/L)	80.8±10.13	165.61±11.45*	124.81±10.62^#^	91.18±13.02^#^^Δ^

*p<0.05 vs Control,

**p<0.01 vs Control,

^#^p<0.05 vs DM,

^##^p<0.01 vs DM,

^Δ^p<0.05 vs LB

**Fig 2:**
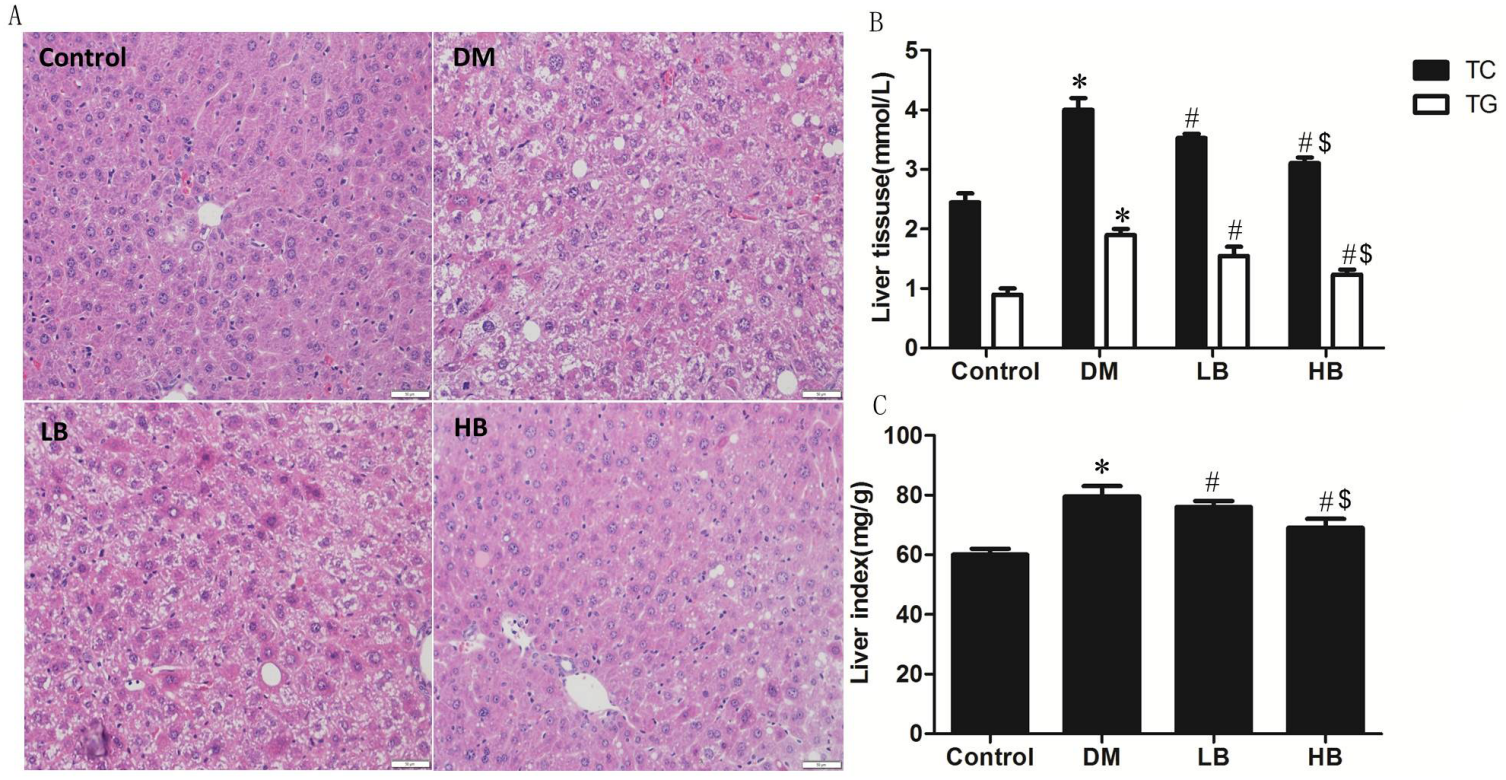
BBR treatment improves liver morphology, liver index and reduces TC and TG in diabetic mice. Liver morphology (A), TC and TG (B) and liver index (C) was determined in Control, DM, LB and HB animals as described in Materials and Methods. Data represent the mean ± SEM (n = 12). *p<0.05 vs Control, ^#^p<0.05 vs DM, ^$^p<0.05 vs LB.

To determine if BBR could improve liver function and morphology in DM animals, the serum levels of the liver enzymes AST and ALT and liver histology were examined. Serum AST and ALT levels were elevated in DM animals compared to controls (Table 2). BBR treatment of DM animals significantly reduced serum AST and ALT in both LB and HB animals. The higher dose of BBR (160mg/kg) in HB treated animals appeared more effective than the lower dose of BBR (40mg/kg) in LB treated animals. The livers of DM animals exhibited cell swelling, vacuolation and infiltration of lymphocytes. BBR treatment significantly reduced cell swelling, vacuolation, and the presence of inflammatory cells (Fig 2A). This was particularly evident in HB animals. TC and TG were elevated in livers of DM animals compared to control (Fig 2B). BBR treatment of DM animals significantly reduced liver TC and TG levels. The higher dose of BBR (160mg/kg) in HB treated animals appeared more effective than the lower dose of BBR (40mg/kg) in LB treated animals. Liver index, expressed as the ratio of liver weight to body weight, was significantly increased in DM animals compared to controls (Fig 2C). BBR treatment of DM animals significantly reduced the liver index compared to DM animals. The higher dose of BBR (160mg/kg) in HB treated animals appeared more effective than the lower dose of BBR (40mg/kg) in LB treated animals. These data indicate that DM animals exhibited a compromised liver function and a lipid metabolism defect which could be partially reversed by BBR treatment.

### BBR treatment attenuates the dysregulation of gluconeogenesis and lipid metabolism in diabetic mice

**Fig 3:**
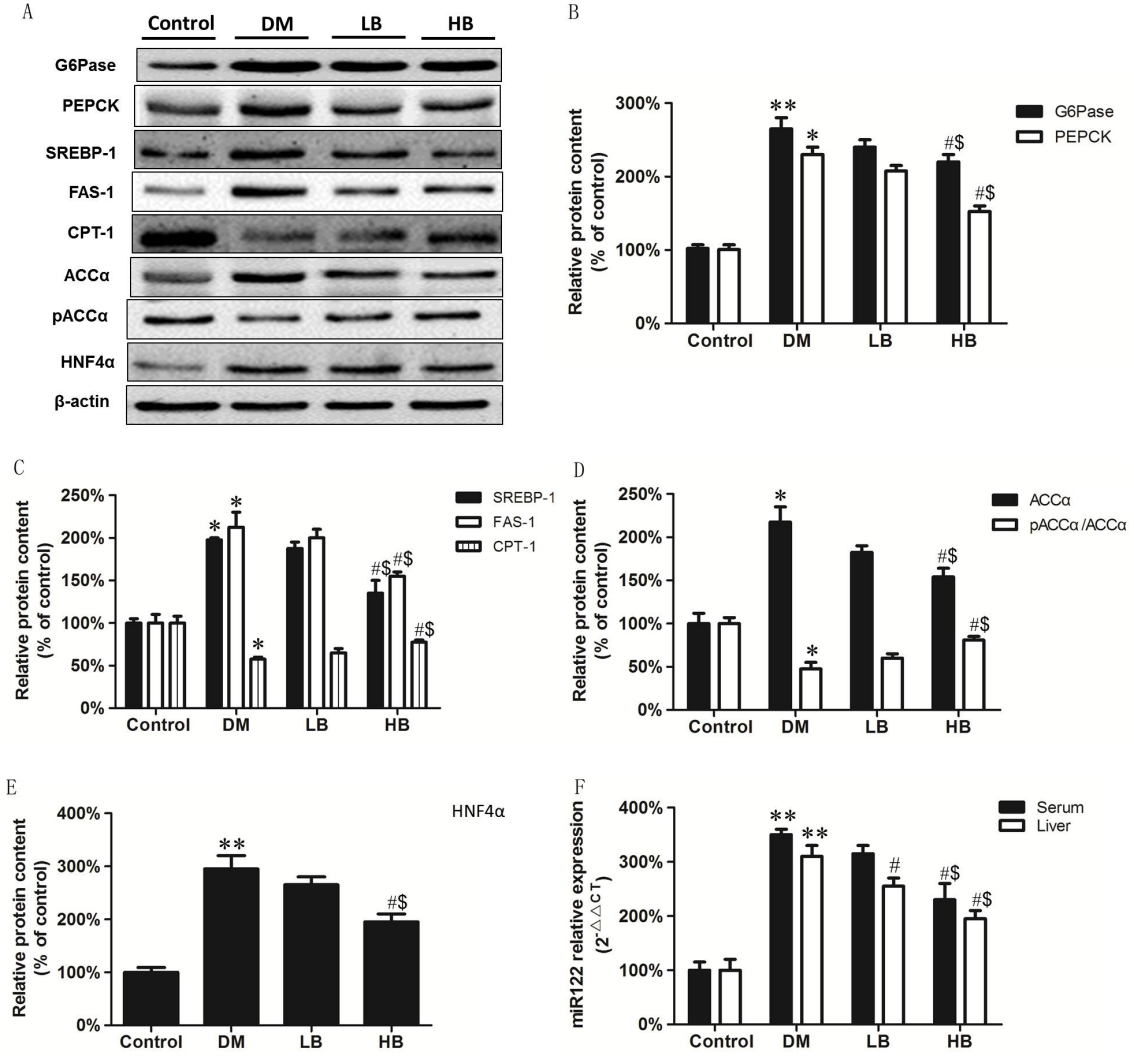
BBR treatment attenuates the alteration in gluconeogenesis and lipid metabolism and expression of HNF-4α and miR122 in liver of DM animals. Expression of G6Pase, PEPCK, SREBP-1, FAS-1, CPT-1, ACCα, pACCα, HNF-4α, β-actin and miR122 were determined in Control, DM, LB and HB animals as described in Materials and Methods. A. A representative blot showing G6Pase, PEPCK, SREBP-1, FAS-1, CPT-1, ACCα, pACCα, HNF-4α and β-actin. B. Relative protein expression of G6Pase and PEPCK. C. Relative protein expression of SREBP-1, FAS-1 and CPT-1. D. Relative protein expression of ACCα and pACCα. E. Relative protein expression of HNF-4α. F. Relative protein expression of serum and liver miR122. Data represent the mean ± SEM (n = 12). *p<0.05, **p<0.01 vs Control; ^#^ p<0.05 vs DM; ^$^p<0.05 vs LB.

We examined if BBR modulated hepatic gluconeogensis and lipid metabolism enzymes in DM animals. G6Pase and PEPCK are important rate-limiting enzymes in the gluconeogenic process in hepatocytes. Expression of liver G6Pase and PEPCK protein were increased in DM animals compared to controls (Fig 3A and 3B). Liver G6Pase and PEPCK protein expression was reduced 19% and 28%, respectively, in HB animals compared to DM animals. SREBP-1 is a major transcriptional regulator of lipogenesis. FAS plays a central role in catalyzing the conversion of acetyl-CoA and malonyl-CoA to palmitate. CPT-1 is a key protein for the control of mitochondrial β-oxidation. Expression of SREBP-1 and FAS-1 were increased and expression of CPT-1 reduced in DM animals compared to controls (Fig 3A and 3C). Liver SREBP-1 and FAS-1 protein expression was reduced 31% and 27%, respectively, in HB animals compared to DM animals. In addition, liver CPT-1 protein expression was increased 36% in HB animals compared to DM animals. ACCα catalyzes the carboxylation of acetyl-CoA to form malonyl-CoA and leads to hepatic TG synthesis and accumulation. Phosphorylation ACCα results in inactivation of ACCα. Total ACCα protein levels were increased and the ratio of pACCα/ACCα reduced in DM animals compared to controls (Fig 3A and 3D). Thus, the amount of activated ACCα was increased in liver of DM animals. Liver ACCα protein levels were reduced 71% in HB animals compared to DM animals. In addition, the levels of pACCα/ACCα were elevated 23% in HB animals compared to DM animals. The above data indicates that BBR treatment could attenuate the dysregulation of gluconeogenesis and lipid metabolism in DM animals.

To further explore key targets of the BBR-mediated improvement of gluconeogenesis and lipid metabolism in DM animals, HNF-4α protein expression in liver and the serum and liver miR122 levels were examined. HNF4α protein expression was significantly increased in the liver of DM animals compared to control (Fig 3E). HNF4α protein expression was reduced 21% and 97% in LB and HB animals, respectively, compared to DM animals. MiR122 levels were increased 2.8-fold in liver and 3.4-fold in serum of DM animals compared to control (Fig 3F). MiR122 levels were reduced 119% in serum and 108% in liver of HB animals compared to DM animals. In addition, miR122 levels were reduced 47% in liver of LB animals compared to DM animals. The above data indicated that BBR treatment attenuated the increased expression of HNF-4α and miR122 in liver of DM animals.

### BBR reverses the alteration of Gluconeogenic and lipid metabolism in palmitate (PA)-incubated HepG2 cells

**Fig 4:**
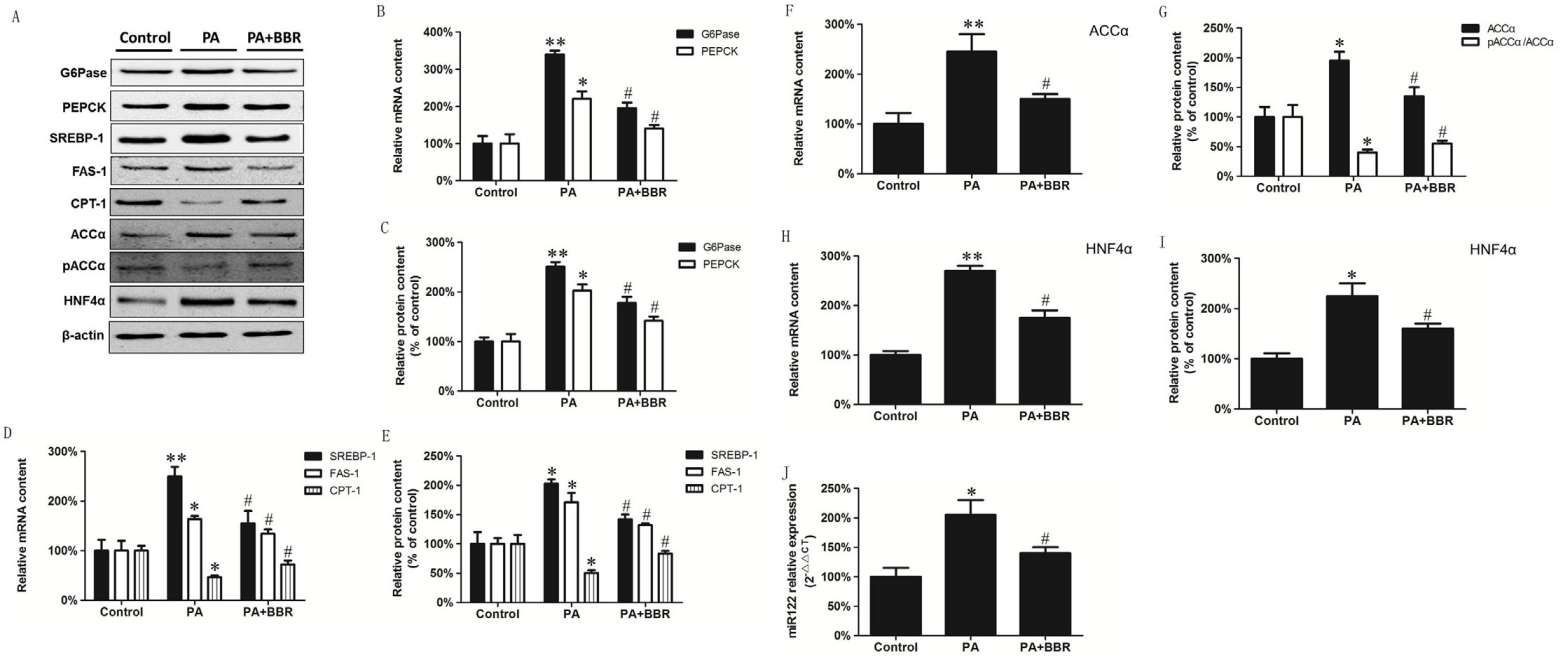
BBR treatment attenuates the alteration in gluconeogenesis and lipid metabolism and expression of HNF-4α and miR122 in PA-treated HepG2 cells. HepG2 cells were incubated in the absence or presence of 0.3 mM PA or 0.3 mM PA with 10 μM BBR for 24 h and expression of G6Pase, PEPCK, SREBP-1, FAS-1, CPT-1, ACCα, pACCα, HNF-4α, β-actin and miR122 were determined as described in Materials and Methods. A. A representative blot showing G6Pase, PEPCK, SREBP-1, FAS-1, CPT-1, ACCα, pACCα, HNF-4α and β-actin. B. mRNA expression of G6Pase and PEPCK. C. Relative protein expression of G6Pase and PEPCK. D. mRNA expression of SREBP-1, FAS-1 and CPT-1. E. Relative protein expression of SREBP-1, FAS-1 and CPT-1. F. Relative protein expression of ACCα and ACCα/pACCα. G. mRNA expression of ACCα. H. mRNA expression of HNF-4α. I. Protein expression of HNF-4α. J. miR-122 expression in HepG2 cells. Data represent the mean ± SEM (n = 12). *p<0.05, **p<0.01 vs Control; ^#^p<0.05 vs PA-treated HepG2 cells.

HepG2 cells incubated with palmitate (PA) were used as a separate model to confirm the observation of altered hepatic gluconeogenesis and lipid metabolism in DM animals. HepG2 cells were incubated in the absence or presence of 0.3 mM PA (S2 Fig) or 0.3 mM PA with 10 μM BBR (S1 Fig) for 24 h and expression of gluconeogenic and lipid metabolism enzymes determined. MRNA and protein expression of G6Pase and PEPCK were significantly increased in PA treated HepG2 cells compared to controls (Fig 4A, 4B and 4C). In addition, mRNA and protein expression of SREBP-1 and FAS were significantly increased in PA treated HepG2 cells compared to controls (Fig 4A, 4D and 4E). The presence of BBR attenuated the elevation of mRNA and protein expression of G6Pase, PEPCK, SREBP-1 and FAS (Fig 4A–4E). In contrast, CPT-1 mRNA and protein expression was significantly decreased in PA treated HepG2 cells compared to controls (Fig 4A, 4D and 4E). The presence of BBR increased CPT-1 protein and mRNA expression compared to PA-incubated HepG2 cells (Fig 4A, 4D and 4E). The total amount of ACCα mRNA and protein expression was increased and the ratio of pACCα/ACCα was decreased in PA-incubated HepG2 cells compared to controls indicating that the amount of activated ACCα was increased in these cells (Fig 4A, 4F and 4G). The presence of BBR attenuated ACCα mRNA and protein expression and increased the ratio of pACCα/ACCα in PA-incubated HepG2 cells. This data indicated that BBR treatment could attenuate the alteration in heaptic gluconeogenesis and lipid metabolism enzymes induced by PA incubation.

To further explore key targets of the BBR-mediated improvement of gluconeogenesis and lipid metabolism in PA-treated HepG2 cells, HNF-4α mRNA and protein expression and miR122 levels were examined. Expression of HNF-4α mRNA and protein were elevated in PA-incubated HepG2 cells compared to control (Fig 4A, 4H and 4I). The presence of BBR attenuated this elevation in HNF-4α mRNA and protein expression in PA-incubated HepG2 cells. MiR122 levels were significantly increased in PA-incubated HepG2 cells compared to controls (Fig 4J). The presence of BBR attenuated this elevation in miR122 in PA-incubated HepG2 cells. Thus, the alterations in expression of gluconeogenic and lipid metabolism enzymes and changes in the level of hepatic HNF-4α and miR122 observed in DM mice were similar to that observed in HepG2 cells treated with PA. In addition, BBR treatment attenuated the alteration of gluconeogenic and lipid metabolism enzymes and in HNF-4α and miR122 levels in DM mice and HepG2 cells treated with PA in a similar manner. These data suggested that the antidiabetic gluconeogenic and lipid metabolism effects of BBR may be related to its action on the expression of HNF-4α and miR122.

### BBR regulates the expression of hepatic gluconeogenic and lipid metabolism genes through HNF-4α and miR122

**Fig 5:**
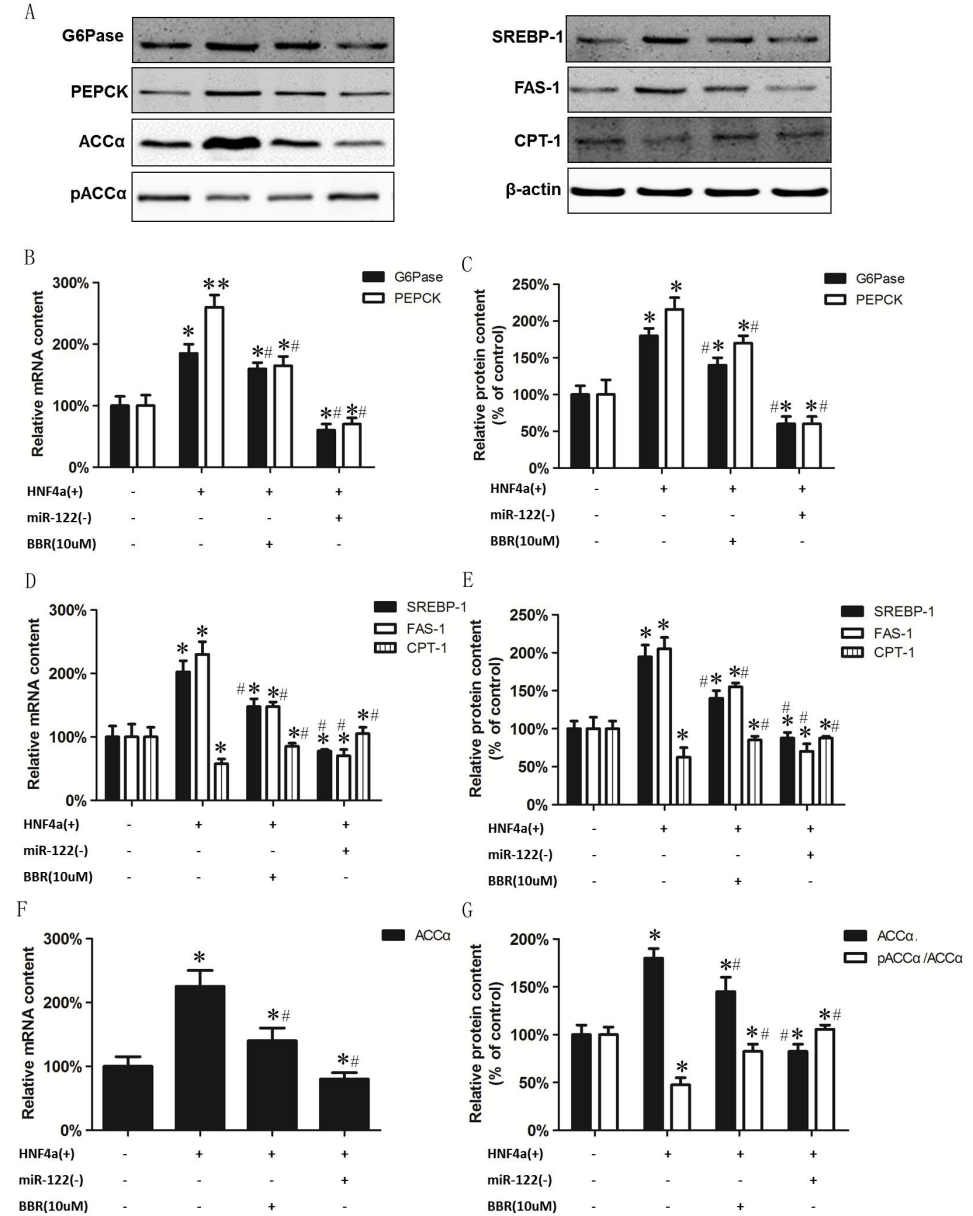
BBR regulates expression of hepatic gluconeogenesis and lipogenic enzymes through HNF4α. HepG2 cells were incubated in the absence or presence of HNF-4α plasmid (HNF-4α(+)) or HNF-4α plasmid plus 10 μM BBR (BBR 10 μM) or HNF-4α plasmid plus miR122 inhibitor (miR122(-)) and the expression of gluconeogenic and lipid metabolism enzymes examined. A. A representative blot showing G6Pase, PEPCK, SREBP-1, FAS-1, CPT-1, ACCα, pACCα and β-actin. B. Protein expression of G6Pase and PEPCK. C. mRNA expression of G6Pase and PEPCK. D. protein expression of SREBP-1, FAS-1 and CPT-1. E. mRNA expression of SREBP-1, FAS-1 and CPT-1. F. protein expression of ACCα and ACCα/pACCα. G. mRNA expression of ACCα. Data represent the mean ± SEM (n = 5). *p<0.05 vs Control; #p<0.05 vs HNF4α(+).

The mechanism of the BBR-mediated regulation of gluconeogenic and lipid metabolism enzymes through HNF-4α and miR122 was examined. HepG2 cells were incubated in the absence or presence of HNF-4α plasmid or HNF-4α plasmid plus 10 μM BBR or HNF-4α plasmid plus miR122 inhibitor and the expression of gluconeogenic and lipid metabolism enzymes examined (S3 Fig). Expression of HNF-4α in HepG2 cells increased mRNA and protein expression of PEPCK and G6Pase compared to controls (Fig 5A, 5B and 5C). Treatment of cells expressing HNF-4α with BBR attenuated the elevated mRNA and protein expression of PEPCK and G6Pase. Expression of HNF-4α in HepG2 cells increased mRNA and protein expression of SREBP-1, FAS, ACCα and reduced mRNA and protein expression of CPT-1 and pACCα compared to controls (Fig 5A, 5D, 5E, 5F and 5G). Treatment of cells expressing HNF-4α with BBR attenuated the increased mRNA and protein expression of SREBP-1, FAS, ACCα and increased mRNA and protein expression of CPT-1 and pACCα. Treatment of cells expressing HNF-4α with miR122 inhibitor attenuated the elevated mRNA and protein expression of PEPCK and G6Pase similar to that of BBR (Fig 5A, 5B and 5C). In addition, treatment of cells expressing HNF-4α with miR122 inhibitor attenuated the increased mRNA and protein expression of SREBP-1, FAS, ACCα and increased mRNA and protein expression of CPT-1 and pACCα similar to that of BBR (Fig 5A, 5D, 5E, 5F and 5G). These results indicate that the effect of BBR on the expression of gluconeogenic and lipid metabolism enzymes may occur through modulation of HNF-4α expression. In addition, the HNF-4α mediated expression of hepatic gluconeogenic and lipid metabolism enzymes are directly regulated by miR122.

**Fig 6:**
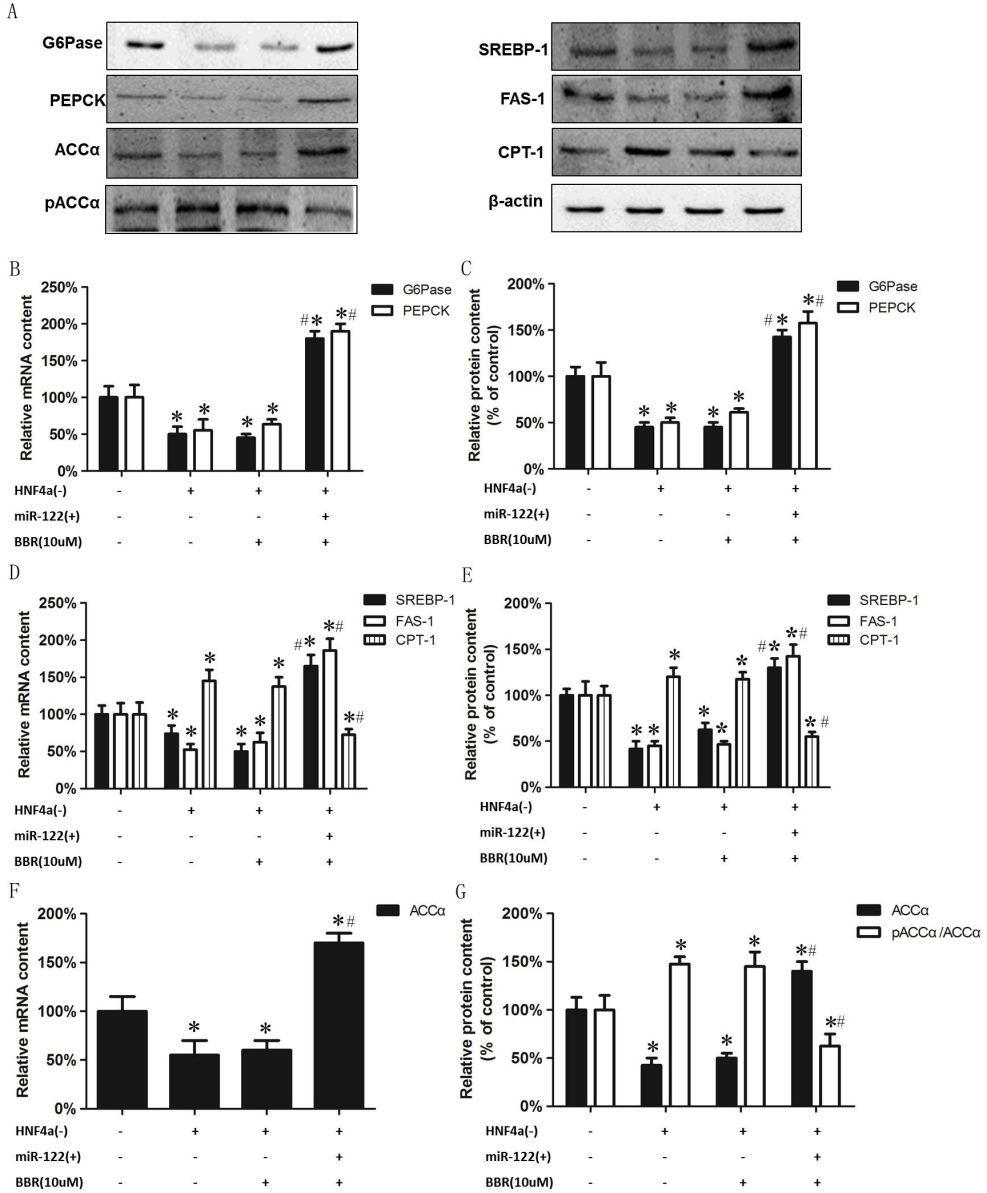
The BBR mediated of expression of hepatic gluconeogenesis and lipogenic genes is not regulated through miR122. HepG2 cells were incubated in the absence or presence of HNF-4α siRNA (HNF-4α(-)) or HNF-4α siRNA plus 10 μM BBR (BBR 10 μM) or HNF-4α siRNA plus miR122 plasmid (miR122(+)) and the expression of gluconeogenic and lipid metabolism enzymes examined. A. A representative blot showing G6Pase, PEPCK, SREBP-1, FAS-1, CPT-1, ACCα, pACCα and β-actin. B. Protein expression of G6Pase and PEPCK. C. mRNA expression of G6Pase and PEPCK. D. protein expression of SREBP-1, FAS-1 and CPT-1. E. mRNA expression of SREBP-1, FAS-1 and CPT-1. F. protein expression of ACCα and ACCα/pACCα. G. mRNA expression of ACCα. Data represent the mean ± SEM (n = 5). *p<0.05 vs Control; #p<0.05 vs HNF4α(-).

To further explore the role of miR122 in the BBR-mediated effect on expression of gluconeogenic and lipid metabolism enzymes, HepG2 cells were incubated in the absence or presence of HNF-4α siRNA (HNF-4α(-)) or HNF-4α siRNA plus 10 uM BBR (BBR(10uM) or HNF-4α siRNA plus 10 uM BBR plus miR122 mimic (miR122(+)) and the expression of gluconeogenic and lipid metabolism enzymes examined. Knockdown of HNF-4α in HepG2 cells reduced mRNA and protein expression of PEPCK, G6Pase, FAS-1 and SREBP-1 (Fig 6A, 6B, 6C, 6D and 6E). The presence of BBR did not alter the reduced mRNA and protein expression of PEPCK and G6Pase mediated by knockdown of HNF-4α in HepG2 cells. In contrast, miRNA122 mimic increased mRNA and protein expression of PEPCK, G6Pase, FAS-1 and SREBP-1 to levels greater than that of control cells. Knockdown of HNF-4α in HepG2 cells increased CPT-1 protein and mRNA expression and the presence of BBR did not alter this (Fig 6A, 6D and 6E). In contrast, miRNA122 mimic decreased CPT-1 mRNA and protein expression to below control levels. Knockdown of HNF-4α in HepG2 cells reduced ACCα protein and mRNA expression and increased the ratio of pACCα/ACCα and the presence of BBR did not alter this (Fig 6A, 6F and 6G). In contrast, miRNA122 mimic increased mRNA and protein levels of ACCα and decreased the ratio of pACCα/ACCα. These data indicate that miR122 is a critical regulator in the downstream pathway of HNF-4α in the regulation of hepatic gluconeogenesis and lipid metabolism in HepG2 cells. In addition, the effect of BBR on hepatic gluconeogenesis and lipid metabolism is mediated through HNF-4α and is regulated downstream of miR122.

## Discussion

The current study was designed to explore the mechanism of the anti-diabetic effect of BBR. Our results indicate that BBR improves hepatic glucose and lipid metabolism by inhibiting the HNF-4α signaling pathway. In addition, activation of HNF-4α leads to the development of a gluconeogenesis and lipid metabolism disorder through miR122. The data strongly support a beneficial role of BBR in improving glucose and lipid homeostasis and suggest that the HNF-4α regulated miR122 pathway is a potential therapeutic target for treatment of T2D with accompanying dyslipidemia.

The therapeutic effects of BBR on the regulation of glucose and lipid levels in diabetic and dyslipidemic patients are well documented [20,33]. However, the mechanism responsible for these therapeutic effects was unknown. BBR treatment of diabetic rats was shown to improve glucose metabolism through inhibition of hepatic gluconeogenesis [34]. In support of this, we observed inhibition of the gluconeogenesis enzymes PEPCK and G6Pase expression in the livers of T2D mice treated with BBR. In addition, BBR treatment of T2D mice inhibited the expression of liver SREBP-1, a transcription factor required for lipogenic gene expression, and expression of liver FAS-1 a key protein involved in fatty acid synthesis. Moreover, BBR treatment of T2D mice increased expression of liver CPT-1 indicating that BBR may upregulate fatty acid β-oxidation. Finally, BBR-treatment of T2D mice elevated liver pACCα thus inhibiting ACCα, a key enzyme in lipid synthesis. We observed the identical effects of BBR on gluconeogenesis and lipid metabolism enzymes in PA-incubated HepG2 cells. These *in vivo* and *in vitro* observations suggest that BBR treatment decreases gluconeogenesis and lipogenesis while simultaneously increasing fatty acid oxidation in livers of T2D mice.

Our observations suggest that HNF-4α is a key target of the BBR-mediated regulation of hepatic gluconeogenesis and lipid metabolism. Expression of liver HNF-4α was inhibited by BBR in both liver of T2D mice and in PA-incubated HepG2 cells. These observations are consistent with our previous study in liver of diabetic rats [13]. In the current study, expression of HNF4α in PA-incubated HepG2 cells increased expression of the hepatic gluconeogenesis enzymes PEPCK and G6Pase and SREBP-1, FAS1 and ACCα, key proteins in lipid synthesis and metabolism, and decreased expression of CPT-1 and BBR treatment attenuated these effects. In addition, expression of hepatic gluconeogenesis and lipid metabolism enzymes were unaltered by BBR treatment in PA-incubated HepG2 cells with knockdown of HNF-4α. These data suggest that the BBR-mediated effect on gluconeogenesis and lipid metabolism is due to the inhibitory action of BBR on the expression of HNF-4α.

We examined if other factors downstream of HNF-4α were involved in the dual effect of BBR on regulation of gluconeogenesis and lipid metabolism. miR-122 is a predominant microRNA in the liver and expression of miR122 was shown to be regulated by HNF4α through its binding to the miR122 promoter in both Huh7 cells and in mouse liver [15]. MiR-122 promotes lipogenesis directly through up-regulation of the expression of lipogenic genes, initially activating SREBP-1c, and subsequently FAS and ACC1 [35]. MiR122 was shown to downregulate the expression of the Cpt1 gene as well as the rate of fatty acid β-oxidation [35]. Mice with liver specific miR122 gene deletion exhibited reduced fatty acid and cholesterol levels [35]. In addition, the expression level of miR122 was elevated in ob/ob mice [36]. Thus, miR-122 may play a role in the accumulation of hepatic triglycerides by promoting lipogenesis and inhibiting fatty acid β-oxidation [35,37]. In the current study, BBR-treatment inhibited HNF4α mRNA and protein expression and reduced expression of miR122 and this was associated with reduction in gluconeogenesis and lipogenesis gene and protein expression in T2D mice and in PA-incubated HepG2 cells. The effect of HNF-4α expression on hepatic gluconeogenesis and lipid metabolism were attenuated by miR122 inhibitor. Interestingly, BBR treatment did not alter gene and protein expression of glucose and lipid metabolism enzymes in cells expressing miR122 with knockdown of HNF-4α. These data further indicate that the dual effect of BBR on hepatic glucose and lipid homeostasis is directly regulated through modulation of HNF-4α expression and not miR122.

## Conclusion

To our knowledge, this is the first study to investigate the effect of BBR on both HNF-4α and miR122 in the regulation of hepatic gluconeogenesis and lipid metabolism in T2D. Our data suggest that BBR exhibits a dual effect on maintenance of both glucose and lipid homeostasis through HNF-4α regulated miR-122 expression. In addition, we suggest that the HNF-4α regulated miR122 pathway may be a key drug target for maintenance of glucose and lipid homeostasis in T2D.

## Supporting Information

S1 Fig Berberine reduces cell viability. HepG2 cells were incubated with various concentrations of Berberine and cell viability determined after 24h. Data represent the mean±S.E.M. (n = 6). *P<0.05, **P<0.01vs control.
(TIF)

S2 Fig Palmitate reduces cell viability. HepG2 cells were incubated with various concentrations of Palmitate and cell viability determined after 24h. Data represent the mean±S.E.M. (n = 6). *P<0.05, **P<0.01vs control.
(TIF)

S3 Fig Transfection efficiency test. A. HNF-4α expression and microRNA expression in HepG2 cells (200 x magnification). B. HNF-4α mRNA levels in HepG2 cells expressing (HNF-4α(+)) (solid bars) or with knock down of HNF-4α (HNF-4α(-)) (open bars). C. Protein expression of HNF-4α in HepG2 cells expressing (HNF-4α(+)) (solid bars) or with knock down of HNF-4α (HNF-4α(-)) (open bars). D. Expression of miR-122 in HepG2 cells in the absence (solid bar) presence (open bar) of miR-122 inhibitor. *p<0.05 vs Control, **p<0.01 vs Control.
(TIF)
