# Supplementary figures and images for: Berberine Attenuates Development of the Hepatic Gluconeogenesis and Lipid Metabolism Disorder in Type 2 Diabetic Mice and in Palmitate-Incubated HepG2 Cells through Suppression of the HNF-4α miR122 Pathway
Source: PLoS One. 2016 Mar 24;11(3):e0152097. doi: 10.1371/journal.pone.0152097 (PMC4806913; doi:10.1371/journal.pone.0152097)

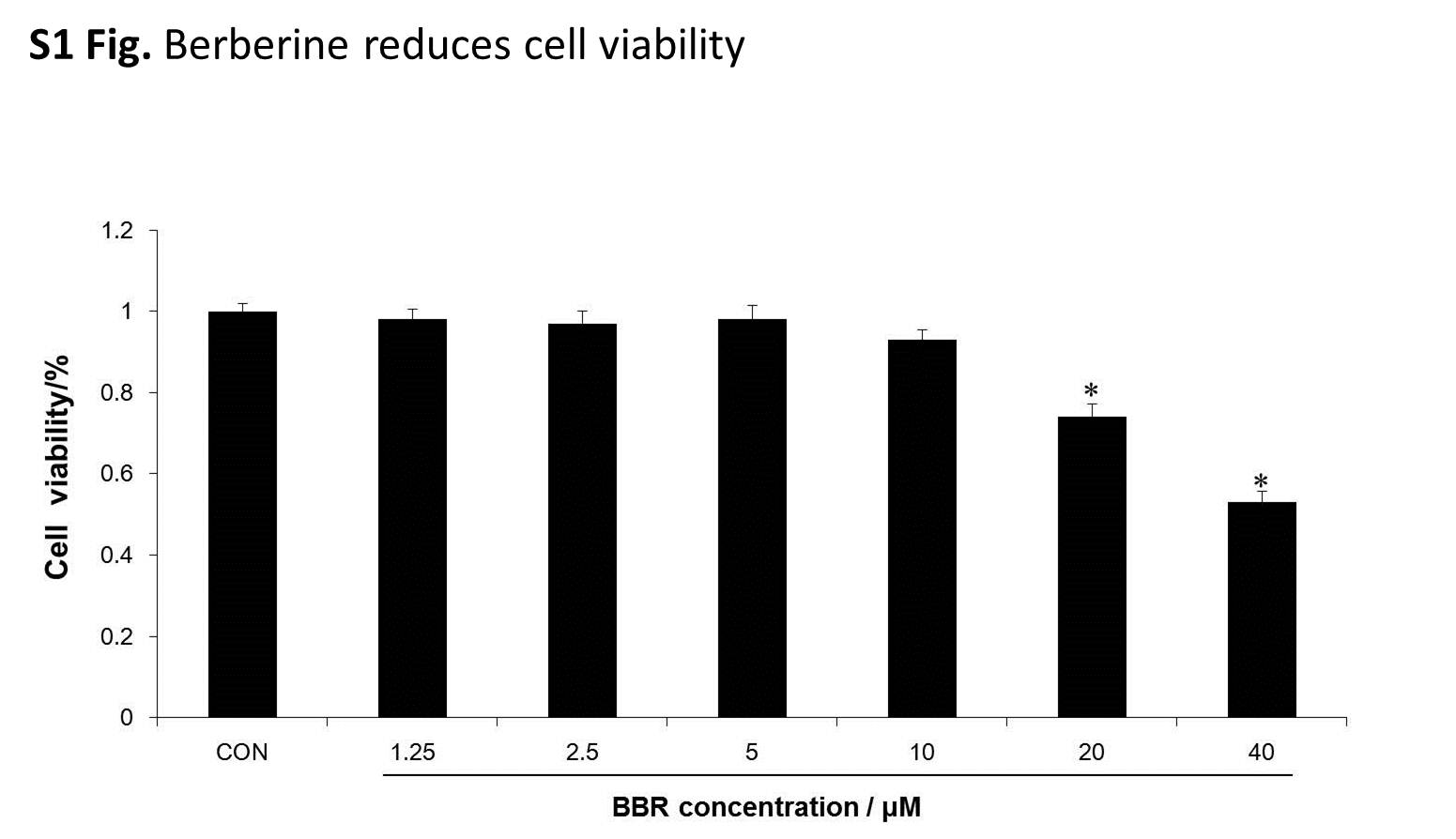

Supplement: S1 Fig — HepG2 cells were incubated with various concentrations of Berberine and cell viability determined after 24h. Data represent the mean±S.E.M. (n = 6). *P<0.05, **P<0.01vs control. (TIF) [file pone.0152097.s001.tif]

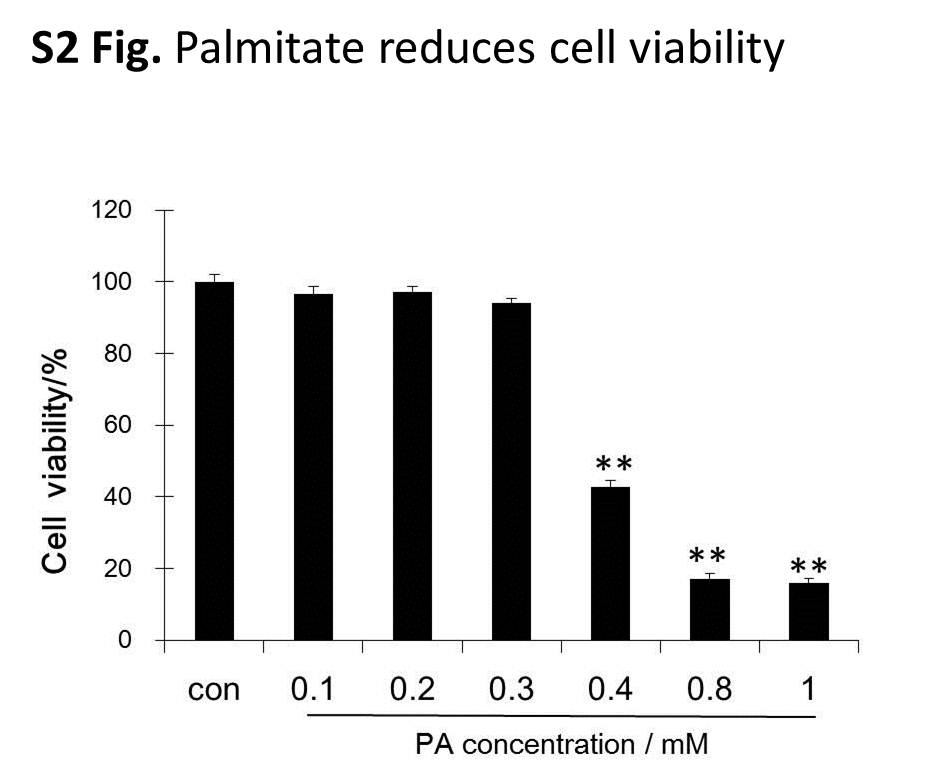

Supplement: S2 Fig — HepG2 cells were incubated with various concentrations of Palmitate and cell viability determined after 24h. Data represent the mean±S.E.M. (n = 6). *P<0.05, **P<0.01vs control. (TIF) [file pone.0152097.s002.tif]

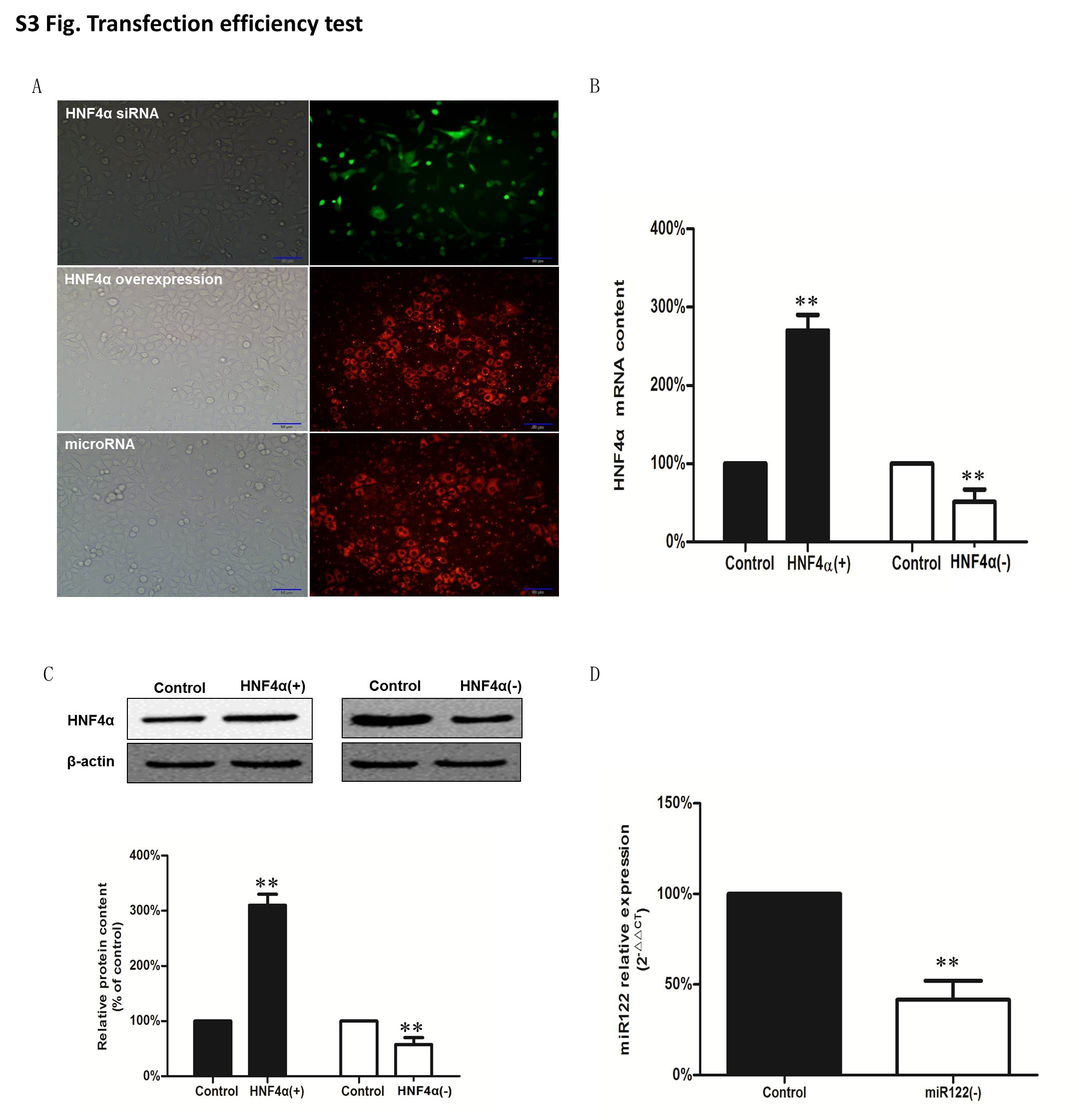

Supplement: S3 Fig — A. HNF-4α expression and microRNA expression in HepG2 cells (200 x magnification). B. HNF-4α mRNA levels in HepG2 cells expressing (HNF-4α(+)) (solid bars) or with knock down of HNF-4α (HNF-4α(-)) (open bars). C. Protein expression of HNF-4α in HepG2 cells expressing (HNF-4α(+)) (solid bars) or with knock down of HNF-4α (HNF-4α(-)) (open bars). D. Expression of miR-122 in HepG2 cells in the absence (solid bar) presence (open bar) of miR-122 inhibitor. *p<0.05 vs Control, **p<0.01 vs Control. (TIF) [file pone.0152097.s003.tif]
